# Common Variants within *MECP2* Confer Risk of Systemic Lupus Erythematosus

**DOI:** 10.1371/journal.pone.0001727

**Published:** 2008-03-05

**Authors:** Amr H. Sawalha, Ryan Webb, Shizhong Han, Jennifer A. Kelly, Kenneth M. Kaufman, Robert P. Kimberly, Marta E. Alarcón-Riquelme, Judith A. James, Timothy J. Vyse, Gary S. Gilkeson, Chan-Bum Choi, R. Hal Scofield, Sang-Cheol Bae, Swapan K. Nath, John B. Harley

**Affiliations:** 1 Department of Medicine, University of Oklahoma Health Sciences Center, Oklahoma City, Oklahoma, United States of America; 2 United States Department of Veterans Affairs Medical Center, Oklahoma City, Oklahoma, United States of America; 3 Arthritis and Immunology Program, Oklahoma Medical Research Foundation, Oklahoma City, Oklahoma, United States of America; 4 Division of Clinical Immunology and Rheumatology, Department of Medicine, University of Alabama at Birmingham, Birmingham, Alabama, United States of America; 5 Department of Genetics and Pathology, Uppsala University, Uppsala, Sweden; 6 Rheumatology Section, Imperial College, Hammersmith Hospital, London, United Kingdom; 7 Division of Rheumatology, Department of Medicine, Medical University of South Carolina, Charleston, South Carolina, United States of America; 8 Division of Rheumatology, Department of Internal Medicine and the Hospital for Rheumatic Diseases, Hanyang University, Seoul, Republic of Korea; University of Hong Kong, China

## Abstract

Systemic lupus erythematosus (SLE) is a predominantly female autoimmune disease that affects multiple organ systems. Herein, we report on an X-chromosome gene association with SLE. Methyl-CpG-binding protein 2 (*MECP2*) is located on chromosome Xq28 and encodes for a protein that plays a critical role in epigenetic transcriptional regulation of methylation-sensitive genes. Utilizing a candidate gene association approach, we genotyped 21 SNPs within and around *MECP2* in SLE patients and controls. We identify and replicate association between SLE and the genomic element containing *MECP2* in two independent SLE cohorts from two ethnically divergent populations. These findings are potentially related to the overexpression of methylation-sensitive genes in SLE.

## Introduction

Systemic lupus erythematosus (SLE) is a debilitating autoimmune disease that affects multiple organs and is associated with significant morbidity and mortality. The disease predominantly affects females with a female to male ratio ranging between 4.3–13.6 to 1 [1]. The etiology of SLE remains incompletely understood, although a number of genetic and environmental factors have been implicated. Strong evidence supports an important role for abnormal T cell DNA methylation in the pathogenesis of SLE [2]. The expression of methylation sensitive genes, such as *ITGAL* (CD11a), *TNFSF7* (CD70), *PRF1* (perforin) and *CD40LG* (CD40L), is increased in T cells from SLE patients, similar to normal T cells treated with DNA methylation inhibitors such as 5-azacytidine [3], [4], [5], [6]. Indeed, 5-azacytidine treated T cells are autoreactive in vitro [7], and produce a SLE-like disease upon adoptive transfer into mice [8]. In active SLE T cells, the expression of DNA methyltransferase 1 (DNMT1), the main enzyme that maintains DNA methylation during cell division, is reduced [9], and the promoter sequences of the aforementioned methylation-sensitive genes are hypomethylated [2], [6]. DNA methylation suppresses gene expression via several mechanisms including the inability of transcription factors to bind methylated promoter sequences [10]. Methyl-CpG-binding protein 2 (MECP2) plays a critical role in this process. MECP2 binds methylcytosine residues and recruits histone deacetylase enzymes, which by deacetylating histone residues, increase the charge attraction between DNA and histone proteins and induce a chromatin configuration that is inaccessible for the transcriptional machinery [11]. Further, DNMT1 associates with and seems to require MECP2 in order to maintain DNA methylation [12].

*MECP2* is located on chromosome Xq28 in man and contains four exons. It is ∼76 kb in length and is characterized by the presence of a very large intron 2 (∼60 kb) and a highly conserved 3′ UTR (∼8.5 kb) [13]. The gene encodes a 486 amino acid chromatin-associated protein that consists of three domains; a methyl-binding domain, a transcription repression domain, and a third domain on the C-terminal region that has not been fully functionally characterized [13]. The facts that DNA methylation sensitive genes are overexpressed in SLE [2], and that MECP2 is critical in the transcriptional suppression of methylation sensitive genes [11], make *MECP2* an attractive candidate gene for SLE. Using a candidate gene approach and a case-control genetic association study, we report herein on the association of the *MECP2* gene region with SLE in two independent cohorts of SLE patients and controls.

## Results

**Figure 1:**
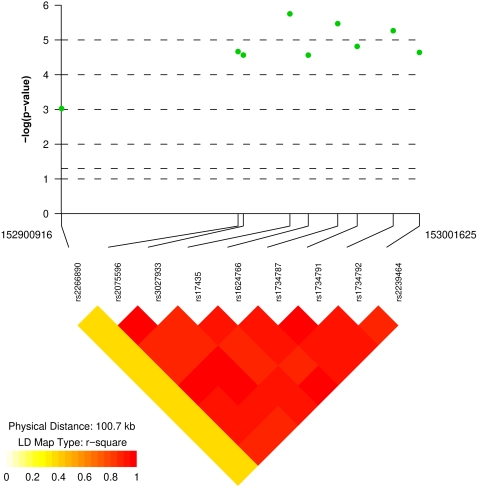
Allelic association results and linkage disequilibrium (LD) plot of the chromosome Xq28 region around the *MECP2* gene. The allelic association p values of the SNPs analyzed are shown in the Korean cohort included in this study.

We initially genotyped 628 Korean female SLE patients and 736 healthy female Korean controls across 21 single nucleotide polymorphisms (SNPs) located within or around *MECP2* (Table 1). Nine SNPs had a minor allele frequency of more than 5% in our Korean cohort and were used for further analysis. All 9 SNPs were within expected Hardy-Weinberg proportions in both cases and controls (Table 2). Eight out of the nine SNPs are within the *MECP2* gene and showed significant association with SLE (Table 2 and Fig. 1). The SNP having the strongest association in the Korean SLE patients is rs17435 (Chi^2^ = 22.83, OR = 1.58, p = 0.0000018) followed by rs1734787 (Chi^2^ = 21.58, OR = 1.55, p = 0.0000034), rs1734792 (Chi^2^ = 20.68, OR = 1.53, p = 0.0000054) and rs1734791 (Chi^2^ = 18.70, OR = 1.51, p = 0.000015). The SNPs rs1734787, rs1734792, and rs1734791 are all in linkage disequilibrium with rs17435 (r^2^ = 0.88, 0.92, and 0.86, respectively). We next performed haplotype-based association test using Haploview 3.32 software [14] and WHAP [15]. Three haplotypes (with a frequency of >1%) were identified (Table 3). The haplotype “ACTGCAAA” was identified as a disease risk haplotype with a frequency of 82.3% in SLE patients compared to 75.3% in normal healthy controls (OR = 1.53, p = 0.000013). On the other hand, the haplotype “GGAAATCG” is a protective haplotype with a frequency of 16.8% in SLE patients and 23.4% in normal healthy controls (OR = 0.66, p = 0.000027) (Table 3). Frequencies of the homozygous risk genotypes in the haplotype-forming SNPs were analyzed and summarized in Table 4.

**Table 1:** SNPs genotyped in the *MECP2* region in SLE patients and controls

SNP	Position (bp)	Alleles	Gene
rs2266890	152900916	A/G	*TMEM187*
rs10127175	152937366	A/T	*IRAK1*
rs2075596	152950586	A/G	*MECP2*
rs3027933	152952068	C/G	*MECP2*
rs3027935	152957662	A/G	*MECP2*
rs3027939	152963772	T/C	*MECP2*
rs17435	152965174	T/A	*MECP2*
rs7050901	152967504	T/C	*MECP2*
rs7059306	152968184	A/G	*MECP2*
rs1624766	152970348	A/G	*MECP2*
rs7884370	152976075	T/C	*MECP2*
rs1734787	152978640	A/C	*MECP2*
rs5987201	152983236	A/G	*MECP2*
rs1734791	152984114	T/A	*MECP2*
rs1734792	152994254	A/C	*MECP2*
rs11156611	152997368	A/G	*MECP2*
rs5987204	153000782	A/G	*MECP2*
rs2239464	153001625	A/G	*MECP2*
rs5986954	153002489	T/C	*MECP2*
rs5945175	153011951	T/C	*LOC728653,MECP2*
rs34371500	153850987	C/A	-

**Table 2:** Allele frequencies of SNPs in the *MECP2* gene region genotyped in Korean SLE patients and that have a minor allele frequency of >0.05

SNP	Risk allele	Risk allele frequency*		Chi2	OR (95% CI)	p value	Permutation p value	HWE (p exact)	
		Cases n (%)	Controls n (%)					Cases	Controls
rs2266890	A	804 (72.8)	848 (66.6)	10.94	1.35 (1.13–1.61)	0.0009	0.0029	1.00	0.24
rs2075596	A	1026 (81.7)	1103 (74.9)	18.05	1.49 (1.24–1.80)	0.000022	0.0001	0.59	0.56
rs3027933	C	1025 (81.6)	1103 (74.9)	17.61	1.48 (1.23–1.79)	0.000027	0.0001	0.35	0.56
rs17435	T	1036 (82.5)	1103 (74.9)	22.83	1.58 (1.31–1.90)	0.0000018	0.00001	0.33	0.85
rs1624766	G	1044 (83.1)	1128 (76.6)	17.59	1.50 (1.24–1.82)	0.000027	0.0001	0.15	0.31
rs1734787	C	1032 (82.2)	1101 (74.8)	21.58	1.55 (1.29–1.87)	0.0000034	0.00003	0.28	0.50
rs1734791	A	1032 (82.2)	1109 (75.3)	18.70	1.51 (1.25–1.82)	0.000015	0.00009	0.28	0.28
rs1734792	A	1018 (81.1)	1085 (73.7)	20.68	1.53 (1.27–1.83)	0.0000054	0.00003	0.52	0.78
rs2239464	A	1044 (83.1)	1127 (76.6)	17.94	1.51 (1.25–1.82)	0.000023	0.0001	0.09	0.30

OR, odds ratio; CI, confidence interval; HWE, Hardy-Weinberg equilibrium.

* A total of 628 independent female SLE patients and 736 healthy unrelated female controls were genotyped

**Table 3:** *MECP2* haplotype frequencies in the Korean SLE patients and controls.

									Frequency (%)			
	rs2075596	rs3027933	rs17435	rs1624766	rs1734787	rs1734791	rs1734792	rs2239464	Case	Control	OR (95% CI)	p value
**Haplotype1**	A	C	T	G	C	A	A	A	82.3	75.3	1.53 (1.26–1.85)	0.000013
**Haplotype2**	G	G	A	A	A	T	C	G	16.8	23.4	0.66 (0.54–0.80)	0.000027
**Haplotype3**	G	G	T	G	A	T	C	A	0.9	1.3	0.74 (0.36–1.50)	0.39

OR, odds ratio; CI, confidence interval.

**Table 4:** Frequencies of homozygous risk allele genotypes in Korean SLE patients compared to controls.

SNP	Risk genotype	Risk genotype frequency*		Chi2	OR (95% CI)		p value
		Cases n (%)	Controls n (%)				
rs2075596	AA	421(67.0)	410 (55.7)	18.28	1.62	(1.30–2.02)	0.00002
rs3027933	CC	422 (67.2)	410 (55.7)	18.81	1.63	(1.31–2.03)	0.00001
rs17435	TT	431 (68.6)	412 (56.0)	22.98	1.72	(1.38–2.15)	0.0000016
rs1624766	GG	439 (69.9)	427 (58.0)	20.66	1.68	(1.34–2.11)	0.0000055
rs1734787	CC	428 (68.2)	408 (55.4)	23.10	1.72	(1.38–2.15)	0.0000015
rs1734791	AA	428 (68.2)	412 (56.0)	21.23	1.68	(1.35–2.10)	0.0000041
rs1734792	AA	415 (66.1)	398 (54.1)	20.29	1.66	(1.33–2.06)	0.0000067
rs2239464	AA	440 (70.1)	426 (57.9))	21.70	1.70	(1.36–2.13)	0.0000032

OR, odds ratio; CI, confidence interval.

* A total of 628 independent female SLE patients and 736 healthy unrelated female controls were genotyped

**Figure 2:**
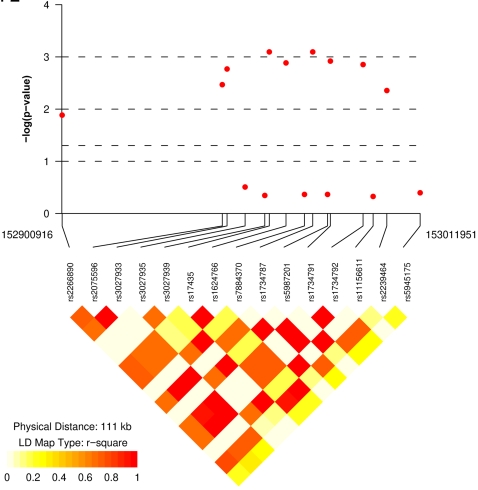
Allelic association results and linkage disequilibrium (LD) plot of the chromosome Xq28 region around the *MECP2* gene. The allelic association p values of the SNPs analyzed are shown in the European-derived cohort included in this study.

To replicate our initial results, we next genotyped 1080 European-derived independent SLE patients and 1080 healthy unrelated controls matched for sex and race using the same 21 SNPs in the *MECP2* region (Table 1). Fifteen SNPs had a minor allele frequency of more than 5% in our European-derived SLE patients and controls and were used for subsequent analysis. All SNPs that were associated with SLE in Korean patients showed significant association with the same risk alleles in the European-derived cohort (Table 5 and Fig. 2). Similarly, the association with these SNPs is confirmed when only analyzing females in the European-derived SLE patients and controls, with the strongest association observed in rs1734787, rs17435, rs1734791, and rs1734792 (p values = 0.0016, 0.0017, 0.0020, and 0.0022, respectively). Our haplotype analysis in female European-derived SLE patients and controls also identified 3 haplotypes with the same risk and protective haplotypes as in the Korean cohort (Table 6). Subset analysis of male European-derived SLE patients and controls was not possible due to small sample size.

**Table 5:** Allele frequencies of *MECP2* SNPs in European-derived SLE patients and controls

SNP	Risk allele	Risk allele frequency*		Chi2	OR (95% CI)		p value	Permutation p value	HWE (p value)	
		Cases n (%)	Controls n (%)						Cases	Controls
rs2266890	A	418 (21.2)	354 (18.0)	6.24	1.22	(1.04–1.43)	0.013	0.068	0.45	0.21
rs2075596	A	371(18.5)	301(15.5)	8.59	1.28	(1.09–1.52)	0.0034	0.02	0.70	1
rs3027933	C	383 (19.1)	308 (15.3)	9.83	1.30	(1.10–1.53)	0.0017	0.01	0.72	0.92
rs3027935	G	1879 (93.8)	1865 (93.0)	1.01	1.14	(0.89–1.46)	0.31	0.79	0.03	0.14
rs3027939	G	104 (5.5)	97 (5.0)	0.57	1.12	(0.84–1.48)	0.45	0.92	0.02	0.68
rs17435	T	500 (24.9)	411 (20.5)	11.25	1.29	(1.11–1.49)	0.0008	0.0047	0.27	0.36
rs1624766	G	497 (24.8)	412 (20.5)	10.39	1.28	(1.10–1.48)	0.0013	0.0074	0.48	0.36
rs7884370	G	109 (5.4)	98 (4.9)	0.63	1.12	(0.85–1.48)	0.43	0.91	0.02	0.68
rs1734787	C	387 (19.3)	307 (15.3)	11.15	1.32	(1.12–1.56)	0.0008	0.0048	1	0.92
rs5987201	A	109 (5.4)	98 (4.9)	0.62	1.12	(0.85–1.48)	0.43	0.91	0.02	0.68
rs1734791	A	389 (19.4)	311 (15.5)	10.43	1.31	(1.11–1.54)	0.0012	0.0072	0.91	1
rs1734792	A	388 (19.3)	311 (15.5)	10.27	1.31	(1.11–1.54)	0.0014	0.0078	0.88	1
rs11156611	A	108 (5.4)	98 (4.9)	0.51	1.11	(0.84–1.47)	0.47	0.94	0.02	0.68
rs2239464	A	475 (23.7)	400 (19.9)	8.13	1.24	(1.07–1.45)	0.0044	0.026	0.72	0.27
rs5945175	G	115 (5.7)	103 (5.1)	0.70	1.12	(0.86–1.48)	0.40	0.89	0.85	0.45

OR, odds ratio; CI, confidence interval; HWE, Hardy-Weinberg equilibrium.

* A total of 1080 independent cases and 1080 healthy unrelated controls matched for race and sex were genotyped

**Table 6:** *MECP2* haplotype frequencies in the European-derived female SLE patients and controls.

	rs2075596	rs3027933	rs17435	rs1624766	rs1734787	rs1734791	rs1734792	rs2239464	Frequency (%)		OR (95% CI)	p value
									Case	Control		
**Haplotype1**	A	C	T	G	C	A	A	A	19.0	15.4	1.29 (1.08–1.53)	0.0044
**Haplotype2**	G	G	A	A	A	T	C	G	77.0	81.0	0.81 (0.69–0.95)	0.012
**Haplotype3**	G	G	T	G	A	T	C	A	4.0	4.2	0.96 (0.70–1.34)	0.82

OR, odds ratio; CI, confidence interval.

The impact of hidden population stratification on our association study was assessed by genomic control (GC) by estimating the inflation factor (λ) in the samples. We estimated λ = 1.06 in Korean and 1.08 in European-derived samples, hence no significant population stratification was detected. These results are also corroborated with our population structure estimates; one-population model (homogeneous population) fit better than a two-population model (admixture) for both cohorts.

## Discussion

DNA methylation plays a critical role in tissue differentiation, imprinting, transcriptional suppression of parasitic DNA, silencing of transcriptional “noise”, and X-chromosome inactivation [16]. Utilizing a candidate gene approach, we first identified significant association with *MECP2* SNPs and SLE in a cohort of Korean SLE patients and controls. We next replicated the association with *MECP2* SNPs in an independent cohort of SLE patients and controls of European descent. Indeed, the disease-associated alleles in rs17435, rs1734787, rs1734792, and rs1734791 (T, C, A, and A respectively) have meta-analysis p values of 1.2×10^−8^, 1.6×10^−8^, 3.3×10^−8^, and 7.2×10^−8^ respectively (**Table 7**). Interestingly, the disease associated alleles in these four *MECP2* SNPs are ∼4 times more common in Korean as compared to European-derived controls. This might suggest a possible explanation for the higher frequency of SLE in people of Asian descent as compared to Europeans.

**Table 7:** Meta-analysis for risk alleles in SLE-associated *MECP2* SNPs in Korean and European-derived cohorts

SNP	Risk allele	Heterogeneity P value	Meta-analysis OR (95%CI)		Meta-analysis P value
rs2075596	A	0.25	1.38	(1.22–1.55)	2.8×10-07
rs3027933	C	0.31	1.38	(1.22–1.56)	1.6×10-07
rs17435	T	0.10	1.39	(1.24–1.56)	1.2×10-08
rs1624766	G	0.18	1.36	(1.21–1.52)	1.9×10-07
rs1734787	C	0.2	1.42	(1.26–1.60)	1.6×10-08
rs1734791	A	0.28	1.39	(1.24–1.57)	7.2×10-08
rs1734792	A	0.23	1.40	(1.24–1.58)	3.3×10-08
rs2239464	A	0.13	1.34	(1.20–1.51)	6.0×10-07

OR, odds ratio; CI, confidence interval

MECP2 has been extensively studied in the setting of mental retardation and, particularly, Rett syndrome, an X-linked neurodevelopmental disease that has a cumulative incidence of ∼1/10,000 females by the age of 12 years [17]. In the majority of cases, this syndrome is caused by mutations in the *MECP2* gene [18]. MECP2-deficient mice demonstrate clinical neurological findings similar to those observed in patients with Rett syndrome [19], [20], which can be reversed by MECP2 expression [21]. More recently, mutations in the *MECP2* gene have been recognized in a number of other neuropsychiatric illnesses as well [22]. Identifying MECP2 regulated genes had been a challenge in patients with Rett syndrome [23]. Recent studies suggest that MECP2 binding to DNA is selective and requires A/T sequences adjacent to methylated CG sites [24]. In addition to its role in transcriptional regulation, MECP2 interacts with the RNA-binding protein Y box-binding protein 1 (YB-1) and plays a role in RNA splicing [25].

Another interesting gene that is in close proximity to *MECP2* is *IRAK1* (Interleukin-1 receptor-associated kinase1). Both *MECP2* and *IRAK1* are on the same haplotype block in combined Japanese and Chinese individuals genotyped in the International HapMap Project (www.hapmap.org). Moreover, this haplotype block harbors only *MECP2* and *IRAK1* genes. The pivotal role of *IRAK1* in Toll-like receptor signaling and innate immune response [26] makes this an important candidate gene for SLE.

We report on an X-chromosome association in SLE. A role for an X-chromosome gene in this predominantly female disease has long been anticipated. Male patients with Klinefelter's syndrome (47,XXY) have similar risk to develop SLE compared to females (46,XX) (Scofield RH, et al Arthritis and Rheumatism 2003; 48:S383) . Possible explanations for the suggested gene-dose effect are the presence of a SLE susceptibility gene(s) on the X-chromosome, or the overexpression of an X-chromosome gene as a result of loss of random X-chromosome inactivation, or both. X-chromosome inactivation is largely mediated by DNA methylation [27], and DNA methylation is defective in SLE T cells [2]. Hence, X-chromosome genes in SLE female patients and SLE male patients with Klinefelter's syndrome are available for transcription from both copies on the two X-chromosomes. This mechanism is suggested to explain the observed overexpression of the X-chromosome gene *CD40L* in T cells from female SLE patients [6]. Our findings provide evidence for SLE association with an X-chromosome region harboring two genes that are intimately involved in regulating the expression of methylation-sensitive genes and in innate immune response.

In summary, the finding of strong association between the X-chromosome region harboring *MECP2* and SLE suggests an important role for genetic and epigenetic interactions in the pathogenesis of this disease. This might provide more insight for the predominance of SLE in females and suggests a novel mechanism to explain the observed overexpression of methylation-sensitive genes in SLE T cells and the resulting T cell autoreactivity in SLE patients.

## Materials and Methods

### SLE patients and controls

Korean SLE patients and controls were recruited at the Hospital for Rheumatic Diseases, Hanyang University, Seoul, Korea. All patients were of Korean descent and met the 1997 American College of Rheumatology SLE classification criteria. A total of 628 independent female SLE patients and 736 healthy unrelated female controls were studied. No two SLE patients or two controls are blood relatives to avoid intrafamilial correlation bias.

Another independent cohort of SLE patients of European descent was studied. This cohort consisted of 1080 independent cases and 1080 healthy unrelated controls matched for race and sex and recruited in the SLE genetics studies at the Oklahoma Medical Research Foundation as well as from collaborators in the United States, United Kingdom, and Sweden. This cohort included 928 females and 152 males in each of the case and control groups. All SLE patients met the 1997 American College of Rheumatology SLE classification criteria.

The study was approved by the Institutional Review Boards of Hanyang University Medical Center, University of Oklahoma Health Sciences Center, and the Oklahoma Medical Research Foundation. Participants in the study gave written informed consent for the genotyping.

### Genotyping

Twenty one SNPs within or around the *MECP2* gene (Table 1) were genotyped on an Illumina BeadStation 500GX instrument using Illumina Infinum II genotyping assays following manufacturer's recommendations. The SNPs were selected to cover the entire length of *MECP2* and the immediate genetic regions in both the 5′ and 3′ ends of *MECP2*. SNPs were selected from the published SNP databases (http://www.ncbi.nlm.nih.gov/projects/SNP/). In general, we selected SNPs that have been validated by at least two groups, that have a minor allele frequency of ≥5%, and that had been tested successfully on the Illumina genotyping platform that we used in our study. Genotyping data were only used from samples with a call rate greater than 90% of the SNPs screened (98.05% of the samples). The average call rate for all samples was 97.18%.

### Data analysis

A population-based case-control statistical design was employed. For each tested SNP, the quality of the genotyping data was assessed by predetermined quality control inclusion criteria (MAF>5%, SNP call rate>90%, and HWE p value>0.01 among the controls). A Pearson's Chi square was calculated for the frequency of allele associations in cases and controls. P values of <0.05 were considered statistically significant. Odds ratios were calculated under the assumption of normality. Fisher's exact test was used to test for deviation from Hardy-Weinberg equilibrium in the genotyped SNPs in the cases and controls. Permutation p values were calculated to correct for multiple testing using Haploview 3.32 [14]. Haplotype frequencies were estimated using the expectation–maximization algorithm implemented in Haploview 3.32 [14] and WHAP [15]. Haplotype-based association analysis was used to perform regression-based omnibus haplotype frequency tests and haplotype-specific tests, implemented in WHAP. We also used pair-wise SNP correlation (r^2^) structure between the two populations to identify the minimum haplotype length which could carry the risk of SLE development. To control for possible confounding due to population stratification, we used genomic control (GC) as well as structured association analysis. A panel of 63 randomly chosen “null” SNPs genotyped on the same Illumina SNP platform was used for GC and for estimating hidden population structure.
